# The Cost-Effectiveness of Anemia Treatment for Persons with Chronic Kidney Disease

**DOI:** 10.1371/journal.pone.0157323

**Published:** 2016-07-12

**Authors:** Benjamin O. Yarnoff, Thomas J. Hoerger, Siobhan A. Simpson, Meda E. Pavkov, Nilka R. Burrows, Sundar S. Shrestha, Desmond E. Williams, Xiaohui Zhuo

**Affiliations:** 1 RTI International, Research Triangle Park, North Carolina, United States of America; 2 Centers for Disease Control and Prevention, Atlanta, Georgia, United States of America; 3 Merck Research Laboratories, North Wales, Pennsylvania, United States of America; Kaohsiung Medical University Hospital, TAIWAN

## Abstract

**Background:**

Although major guidelines uniformly recommend iron supplementation and erythropoietin stimulating agents (ESAs) for managing chronic anemia in persons with chronic kidney disease (CKD), there are differences in the recommended hemoglobin (Hb) treatment target and no guidelines consider the costs or cost-effectiveness of treatment. In this study, we explored the most cost-effective Hb target for anemia treatment in persons with CKD stages 3–4.

**Methods and Findings:**

The CKD Health Policy Model was populated with a synthetic cohort of persons over age 30 with prevalent CKD stages 3–4 (i.e., not on dialysis) and anemia created from the 1999–2010 National Health and Nutrition Examination Survey. Incremental cost-effectiveness ratios (ICERs), computed as incremental cost divided by incremental quality adjusted life years (QALYs), were assessed for Hb targets of 10 g/dl to 13 g/dl at 0.5 g/dl increments. Targeting a Hb of 10 g/dl resulted in an ICER of $32,111 compared with no treatment and targeting a Hb of 10.5 g/dl resulted in an ICER of $32,475 compared with a Hb target of 10 g/dl. QALYs increased to 4.63 for a Hb target of 10 g/dl and to 4.75 for a target of 10.5 g/dl or 11 g/dl. Any treatment target above 11 g/dl increased medical costs and decreased QALYs.

**Conclusions:**

In persons over age 30 with CKD stages 3–4, anemia treatment is most cost-effective when targeting a Hb level of 10.5 g/dl. This study provides important information for framing guidelines related to treatment of anemia in persons with CKD.

## Introduction

Chronic anemia, a common complication of kidney function loss [1], confounds chronic kidney disease (CKD) treatment and is associated with increased risk of cardiovascular disease (CVD) morbidity and mortality [2]. Defined as hemoglobin (Hb) level <13.0 g/dl in males and <12.0 g/dl in females [3], anemia in persons with CKD may be due to iron deficiency, erythropoietin deficiency, and erythropoietin hypo-responsiveness [2]. Although major guidelines uniformly recommend iron supplementation and erythropoietin stimulating agents (ESAs) for managing chronic anemia in persons with CKD [3–5], there are differences in the recommended Hb treatment target. Specifically, the Kidney Disease Improving Global Outcomes (KDIGO) guidelines recommend Hb level to be maintained between 9 and 11.5 g/dl [3]; the European Best Practice Guidelines recommend that most persons with CKD achieve a target Hb between 11 and 12 g/dl [4]; the National Institute for Health and Clinical Excellence (NICE) recommends a target range between 10 and 12 g/dl [5]. While, the management of anemia may impose a significant cost burden for persons with CKD, none of these clinical guidelines considered cost or cost-effectiveness analysis.

In this study, using the CKD Health Policy Model [6–9], we explored the most cost-effective Hb target for anemia treatment in persons with CKD stages 3–4, representing persons with estimated glomerular filtration rate (eGFR) <60 ml/min/1.73m^2^ not on dialysis. The model incorporated the potential tradeoff between the benefits of higher Hb targets and the side effects of higher ESA doses needed to achieve them. Incremental costs and quality adjusted life years (QALYs) were assessed for Hb targets of 10 g/dl to 13 g/dl at 0.5 g/dl increments.

## Methods

### Model Overview

The CKD Health Policy Model, a microsimulation model of CKD progression, has been described in detail elsewhere [6–9]. Briefly, the model simulates progression of CKD and its complications in a nationally representative synthetic cohort of adults age ≥ 30 years, created from the National Health and Nutrition Examination Survey (NHANES), through age 90 years or death. The model includes seven states: no CKD, CKD stages 1 through 5, and death. CKD and its stages are defined by both eGFR and the presence of elevated albuminuria (urinary albumin to creatinine ratio ≥30 mg/g) [10]. Model parameters related to CKD progression and its complications were derived from the epidemiological literature, clinical trials, and a previous cost-effectiveness study by Boulware et al [11].

In the present analysis, the CKD Health Policy Model was adapted to simulate 1) decline in Hb level; 2) complications associated with both Hb level and corresponding anemia treatment, such as stroke, myocardial infarction, hypertension, blood transfusion, and non-CVD mortality; and 3) quality of life reductions related to lower Hb level. The model was populated with a synthetic cohort of persons age ≥ 30 years with prevalent CKD stage 3–4 and anemia who were not on dialysis at baseline, created from the 1999–2010 NHANES. The cohort included persons with CKD stage 3 or 4 at baseline because of the steep rise in anemia incidence beginning in CKD stage 3 [1] and does not include persons with stage 5 CKD at baseline, because they are not represented in NHANES. Persons may develop stage 5 CKD over the course of the model simulation and the costs and complications for these persons with stage 5 CKD are considered in the analysis.

Key model parameters related to anemia, anemia treatment, and associated complications were derived from the published literature, as shown in Table 1 and discussed in detail below.

**Table 1:** Key parameters used in the CKD Health Policy Model related to anemia, anemia treatment, complications of anemia and its treatment, and anemia treatment costs in persons with CKD stages 3 and 4.

Parameter	Value	Source
HR for complications per 1 g/dl increase in Hb		
Stroke	0.85	Skali et al. 2011 [12]
Myocardial Infarction	1.00	Palmer et al. 2010 [13]
Hypertension	1.00	Conservative assumption
Non-CVD Mortality	0.93	Koulouridis et al., 2013 [14]
HR for complications per 10,000 unit of epoetin alfa dose		
Stroke	1.60	Koulouridis et al., 2013 [14]
Myocardial Infarction	1.00	Koulouridis et al., 2013 [14]
Hypertension	1.13	Koulouridis et al., 2013 [14]
Non-CVD Mortality	1.25	Koulouridis et al., 2013 [14]
HR for blood transfusion, by Hb (g/dl)		
< 7	1.59	Lawler et al., 2010 [15]
7 to 7.9	1.47	Lawler et al., 2010 [15]
8 to 8.9	1.27	Lawler et al., 2010 [15]
9 to 9.9	1.08	Lawler et al., 2010 [15]
10 to 10.9	Reference	Lawler et al., 2010 [15]
11 to 11.9	0.99	Lawler et al., 2010 [15]
12 to 13	0.98	Lawler et al., 2010 [15]
Probability of blood transfusion for Hb 10 to 10.9 if not receiving other anemia treatment	23%	Lawler et al., 2010 [15]
Probability of blood transfusion for Hb 10 to 10.9 if receiving other anemia treatment	2%	Lawler et al., 2010 [15]
HR for blood transfusion per 10,000 unit epoetin alfa dose	0.73	Koulouridis et al., 2013 [14]
Mean number of blood transfusions per year conditional on having any transfusions	1.80	Lawler et al., 2010 [15]
Utility loss per 1 g/dl decrease in Hb (reference Hb ≥13 g/dl)	0.0114	Finklestein et al., 2009 [16] with mapping function from Ara et al., 2008 [17]
Utility loss from stroke	0.582	Meenan et al., 2007 [18]
Utility loss from myocardial infarction	0.12	Tsevat et al., 1993 [19]
Discount rate for QALYs and costs	3%	Weinstein et al. 1996 [20]
*Cost Parameters*		
Weekly cost per 1,000 units epoetin alfa	$9.87	Medicare Part B Drugs 2012 [21]
Cost of annual dose of intravenous iron	$1744	Cost per unit from Medicare Part B Drugs 2012 [21] Monthly dose from Lu et al., 2010 [22]
Cost of blood transfusion	$2126	Blumberg et al. 1996 [23]

CKD, chronic kidney disease; CVD, cardiovascular disease; Hb, hemoglobin; HR, hazard ratio; QALY, quality-adjusted life years.

### Model Calibration

The model was calibrated by comparing simulated results to the results reported from three large CKD-anemia trials: CHOIR [24], CREATE [25], and TREAT [26]. For this calibration, we first created synthetic population cohorts that matched the characteristics of the actual cohorts in each study by randomly generating populations with characteristics (age, gender, race, diabetes, blood pressure, history of CVD) drawn from the actual baseline distributions of each study. The model was populated with each of the synthetic cohorts and simulations were conducted using the target Hb levels of each trial for the synthetic population matched to that trial. Each trial had a high and a low Hb treatment arm and both were examined in the model calibration. The high Hb targets were 13.5 g/dl in CHOIR, 13 to 15 g/dl in CREATE, and 13 g/dl in TREAT. The low Hb targets were 11.3 g/dl in CHOIR, 10.5 to 11.5 g/dl in CREATE, and rescue treatment if Hb fell below 9 g/dl in TREAT. Simulation results were compared to the actual study outcomes for each trial arm and calibrated to fit: 1) the aggregate results of all six trial-arm cohorts, 2) the aggregate results of the three high-target cohorts and the three low-target cohorts, and 3) the results of each of the six individual trial-arm cohorts. The model was calibrated by adjusting multipliers for the relative risk effects of ESA dosage and Hb levels on mortality, myocardial infarction, and stroke. We used an iterative process to find the best fit based on the calibration criteria.

### Anemia and Anemia Treatment

The model assigns an annual (age year) level of Hb based on the Hb distribution among participants with prevalent CKD stage 3–4 in the 1999–2010 NHANES and individual risk factors: age, race, diabetes, CVD, CKD stage, and eGFR. Annual changes in Hb for a person are driven by changes in risk factors in the absence of treatment. This process is documented in detail in the S1 Appendix.

**Fig 1:**
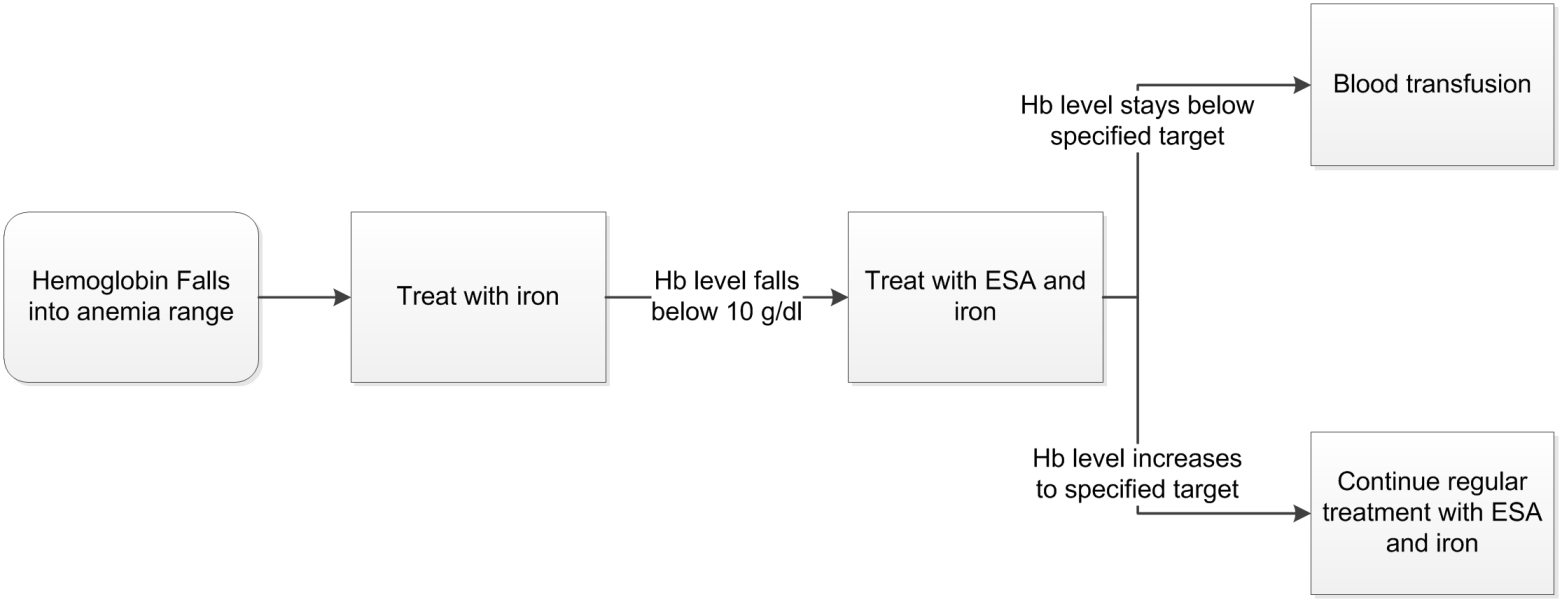
Annualized anemia treatment decision flowchart assumed in the CKD Policy Model.

Annualized anemia treatment is simulated as shown in Fig 1. Persons receive ESA treatment once Hb levels falls below 10 g/dl with incremental doses to restore Hb level to a preset target level. All persons receiving treatment for anemia also received a monthly dose of iron once they become anemic and continue to receive iron once they begin ESA treatment to improve their response to ESA. In practice, this guideline may not be strictly adhered to, so this model assumption represents the perfect world setting. However, relaxing this assumption will not alter results, because we examine costs for incrementally higher Hb targets in persons receiving treatment. We focused on one ESA, epoetin alpha, but the alternative drug, darbepoetin alpha, is a comparable substitute. The model simulates one-year increments, so treatment with ESAs is annualized. If the ESA is ineffective, persons are assigned to receive blood transfusions to increase Hb levels [3]. The necessity of blood transfusions is modeled annually with hazard ratios of not responding to ESA treatment (Table 1). The number of blood transfusions is annualized and on average, persons requiring blood transfusions receive 1.8 each year [15].

To model the ESA dose necessary to achieve a given change in Hb, we fitted a regression equation to data from clinical trials on ESA dose and change in Hb, stratified by baseline Hb. We provide detail on this approach in S1 Appendix. Key treatment parameters are shown in Table 1.

### Complications of Anemia and Anemia Treatment

The model simulates the incidence of complications related to anemia as a function of Hb concentration level and ESA dose based on published findings indicating that higher Hb levels, up to normal levels, are associated with decreased risk of complications, particularly stroke and non-CVD mortality, while large ESA dose is associated with increased risk of these same complications [14, 27–29]. These published findings suggest that there may be a tradeoff between increasing ESA dosage and increasing Hb levels: increased ESA dosage is associated with increased risk of complications, but also increases Hb level which is associated with reduced risk of complications. The model structure incorporates this tradeoff by including hazard ratios for the incidence of stroke, myocardial infarction, hypertension, blood transfusion, and non-CVD mortality associated with Hb concentration and ESA dose. We chose this approach to represent the relationship observed when comparing recent large clinical trials such as CHOIR [24], CREATE [25], and TREAT [26]: health outcomes improved with treatment to low Hb targets but declined to levels at or below placebo for higher Hb targets. The research examining the tradeoff between increasing ESA dosage and increasing Hb levels is new and potentially controversial, so we rigorously tested the sensitivity of our results to these assumptions about side effects of ESA dose. Hazard ratio parameters for Hb concentration and ESA dosage are listed in Table 1.

### Costs of Anemia Treatment

The model simulates annual costs associated with CKD and its complications as well as treatment for anemia. The modeling process for costs of CKD and its complications has been described in detail elsewhere [6–9]. Medical costs for anemia complications are included in the core CKD model. Costs of anemia treatment are for ESA treatment with epoetin alpha, intravenous iron, and blood transfusion (Table 1) and are based on drug prices from the Medicare part B schedule and other published literature [21–23]. Incremental costs associated with treatment regimens were determined by differences in ESA dose and differences in costs from anemia complications. Costs are modeled in 2010 dollars. To incorporate time preferences, costs are discounted 3% annually.

### Quality of Life

The summary effectiveness outcome measure was QALYs, which were calculated as the present value of future life-years, indexed by the person’s quality of life (on a utility scale). Life years are computed from baseline to death or age 90 (model end) and are reduced by premature death due to complications associated with anemia such as stroke and myocardial infarction. The analysis includes persons that progress to stage 5 CKD. Quality of life is reduced by declines in utility associated with anemia complications (stroke and myocardial infarction). Anemia also decreases quality of life by reducing general energy and vitality [16,30]. This direct effect of anemia on quality of life was modeled as a function of Hb concentration relative to a baseline Hb concentration of 13 g/dl. We took average quality of life decrements per 1 g/dl decrease in Hb level from a baseline of 13 g/dl from Finkelstein et al. [16] Because the quality of life measure used in that study was not based on health state utility, we used the mapping function of Ara et al. [17] to convert quality of life to utility measures. The reduction in quality of life for each 1 g/dl decrease in Hb below a baseline level of 13 g/dl is given in Table 1.

### Cost-Effectiveness Ratios

Lifetime costs and QALYs were simulated for each treatment scenario. For each Hb target, incremental QALYs and costs were calculated as the difference in QALYs and costs from the next lower Hb target. The treatment targets ranged between 10 g/dl and 13 g/dl at 0.5 g/dl increments. The scenario of no treatment with ESA serves as the comparison group for a treatment target of 10 g/dl. Incremental cost-effectiveness ratios (ICERs) were computed as incremental cost divided by incremental QALYs for each target Hb. Costs and QALYs were discounted at a 3% annual rate, as recommended by Weinstein et al. [20]. We evaluated costs to the health care system.

We determined whether or not a treatment target is cost-effective following the conventional cost-effectiveness benchmark of $50,000 per QALY [31] (i.e., a treatment target is considered cost-effective if the ICER is below the $50,000 benchmark for the willingness pay). Among the cost-effective treatment targets, the most cost-effective target is the one that has the highest QALYs gained.

### Sensitivity Analyses

To test the sensitivity of our results and conclusions to the choice of parameters for risks, costs, and quality of life effects, we conducted a number of one-way sensitivity analyses by varying key model parameters. Because of the emerging nature of the evidence for the risks associated with ESA dose, it was most important to test the sensitivity of results to these parameters. Therefore, we tested the sensitivity of results to 50% and 100% decreases in the hazard ratios for all-cause mortality, stroke, and hypertension associated with Hb level and ESA. In addition, we examined the impact of changes in several other key model parameters, including; a 100% decrease in the quality of life decrement associated with Hb; ±50% changes in the cost of ESA dose; and ±1 g/dl changes in the Hb level at which anemia treatment is started.

Probabilistic sensitivity analyses (PSA) were conducted by varying hazard ratios for non-CVD mortality, stroke, and hypertension associated with Hb level and ESA dose, the cost of ESA dose, and the quality of life decrement associated with Hb level along distributions reported in the published literature used to generate the main parameters. Table 2 contains the parameters used for this probabilistic sensitivity analysis. The net benefit of each PSA draw was computed as willingness to pay*total QALYs–total costs. We generated cost-effectiveness acceptability curves that examined the fraction of draws for each Hb target that had the highest net benefit for each level of willingness to pay for QALY gains.

**Table 2:** Key parameters for probabilistic sensitivity analysis in the CKD Health Policy Model related to anemia, anemia treatment, complications of anemia and its treatment, and anemia treatment costs in persons with CKD stages 3 and 4.

**Parameters with log-normal distribution**	**Log-normal mean**	**Log-normal SD**	**Source**
HR for complications per 1 g/dl increase in Hb			
Stroke	-0.16	0.08	Skali et al. 2011 [12]
Non-CVD Mortality	-0.07	0.08	Koulouridis et al., 2013 [14]
HR for complications per 10,000 unit of epoetin alfa dose			
Stroke	0.47	0.12	Koulouridis et al., 2013 [14]
Hypertension	0.12	0.05	Koulouridis et al., 2013 [14]
Non-CVD Mortality	0.22	0.10	Koulouridis et al., 2013 [14]
**Parameters with a gamma distribution**	**Alpha**	**Beta**	**Source**
Weekly Cost per 1000 units of epoetin alfa	16	0.617	Medicare Part B Drugs 2012 [21]
Utility loss from a 1 g/dl decrease in Hb (reference group: Hb > = 13 g/dl)	1	0.0114	Finklestein et al., 2009 [16] with mapping function from Ara et al., 2008 [17]

## Results

**Fig 2:**
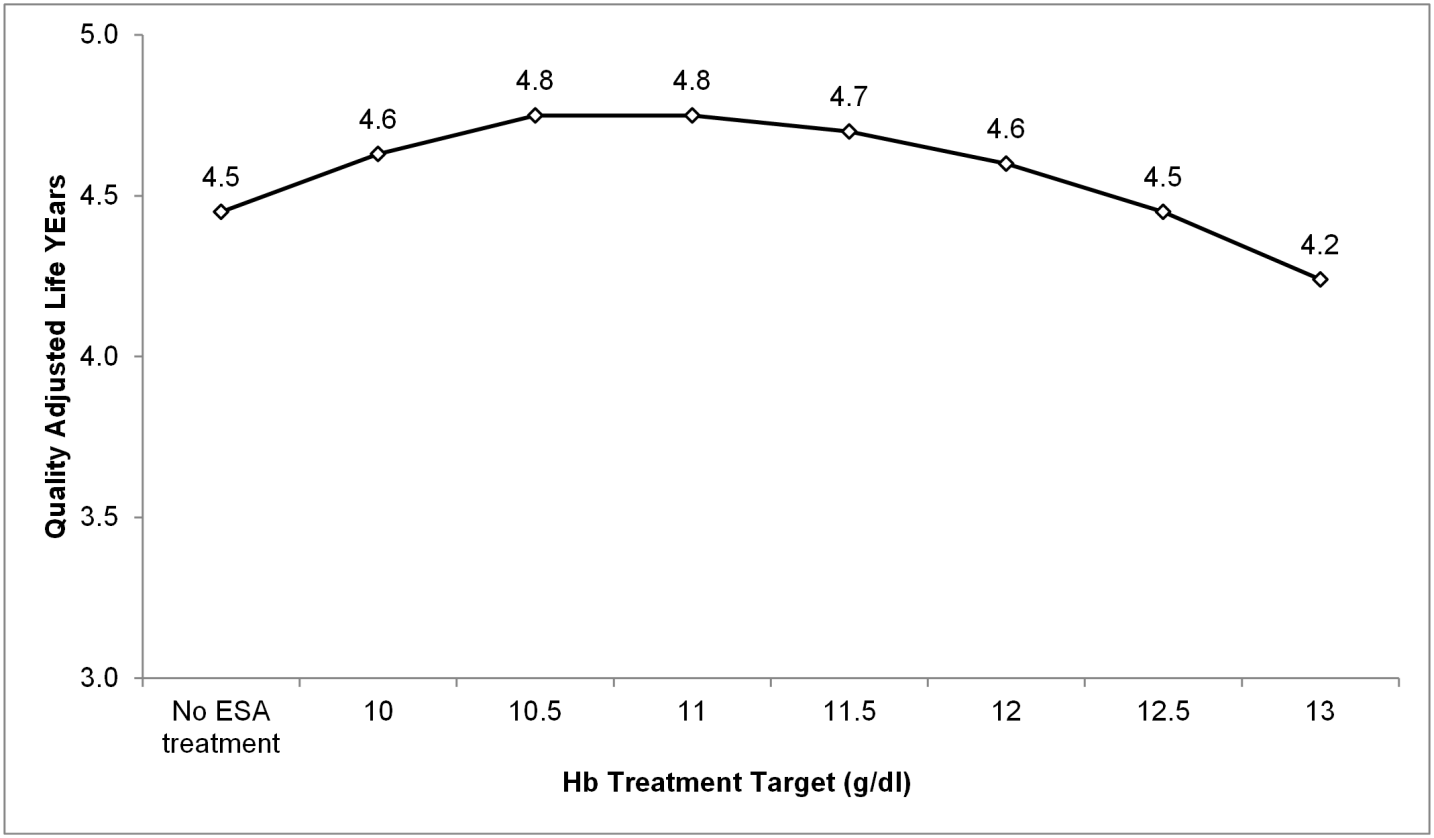
Quality adjusted life years as a function of Hb treatment targets illustrates an inverted U-shaped relationship in persons with CKD stages 3–4. ESA, erythropoietin stimulating agents; Hb, hemoglobin.

Table 3 presents average lifetime medical costs, life years, QALYs, and ICERs from simulations of Hb treatment targets of 10 g/dl to 13 g/dl by 0.5 g/dl increments and no treatment with ESA. Average lifetime medical costs increased from $94,056 for the case with no ESA treatment (only iron) to $140,925 for a treatment with a target Hb of 13 g/dl. ESA dose increased from 439 units for an Hb target of 10 g/dl to 27,145 units for Hb target of 13 g/dl. Life years increased to 6.84 when the treatment target was 10 g/dl and to 6.99 when the treatment target was 10.5 g/dl, but decreased incrementally at higher Hb targets. Increasing the target Hb to 11 g/dl resulted in 0.39 additional life years compared to no ESA treatment, but 0.06 fewer life years than for a target of 10.5 g/dl. Life years continued to fall for treatment targets higher than 11 g/dl, although they remained higher than the no ESA treatment scenario for targets ≥11 g/dl. QALYs increased to 4.63 for a treatment target of 10 g/dl and to 4.75 for a target of 10.5 g/dl or 11 g/dl, and fell for higher Hb targets. QALYs fell for treatment targets higher than 11.5 g/dl, although they remained higher than the no ESA treatment case for targets ≥11.5 g/dl. Fig 2 presents the relationship between Hb target and QALYs, highlighting the inverted U-shaped pattern.

**Table 3:** Incremental cost-effectiveness as a function of anemia treatment targets in persons with CKD stages 3–4.

Hb Target (g/dl)	Lifetime Medical Costs ($)	ESA dose (units/week)	Life Years	QALYs	ICER ($/QALY)
No ESA treatment	94,056 (2,374)	NA	6.54 (0.13)	4.45 (0.09)	
10.0	99,836 (2,438)	439 (13)	6.84 (0.14)	4.63 (0.09)	32,111 (261,513)
10.5	103,733 (2,511)	1,140 (16)	6.99 (0.14)	4.75 (0.09)	32,475 (1,575,585-)
11.0	109,359 (2,568)	4,175 (46)	6.93 (0.14)	4.75 (0.09)	-
11.5	115,987 (2,551)	8,467 (82)	6.80 (0.13)	4.70 (0.09)	Dominated
12.0	123,813 (2,862)	13,674 (117)	6.61 (0.13)	4.60 (0.09)	Dominated
12.5	132,406 (2,889)	19,864 (149)	6.35 (0.12)	4.45 (0.10)	Dominated
13.0	140,925 (3,086)	27,145 (171)	6.01 (0.12)	4.24 (0.10)	Dominated

Indicated values are means. Values in parentheses are standard deviations.

CKD, chronic kidney disease; ESA, erythropoietin stimulating agents; Hb, hemoglobin; ICER, incremental cost-effectiveness ratio; NA, not applicable; QALY, quality-adjusted life years.

The ICER for a 10 g/dl treatment target was $32,111 and the ICER for a 10.5 g/dl target was $32,475 (Table 3). The ICER for an 11 g/dl target was undefined because there was no change in QALYs from a 10.5 g/dl target. Any treatment target above 11 g/dl was a dominated strategy (i.e. worsened both health and cost outcomes) because these higher Hb targets increased medical cost and decreased QALYs.

### Sensitivity Analyses

One-way sensitivity analyses demonstrated some variation in the treatment target at which QALYs began to decline (Table 4). QALYs decreased incrementally at treatment targets ≥11 g/dl in a sensitivity analysis reducing the hazard ratio of stroke for ESA dosage. When the hazard ratio of non-CVD mortality for ESA dosage was reduced by 50%, a decline in QALYs was observed at treatment target ≥11.5 g/dl. When the hazard ratio was reduced by 100%, QALYs declined at treatment targets ≥12.5 g/dl. In additional sensitivity analyses, reducing the hazard ratio of hypertension for ESA dosage, reducing the QALY decrement from decreased Hb, and varying the cost of ESA, QALYs were incrementally reduced at treatment targets ≥10.5 g/dl. In the sensitivity analysis initiating ESA therapy when Hb reached 9 g/dl, QALYs incrementally declined at treatment targets ≥10 g/dl, and in sensitivity analysis initiating ESA therapy when Hb reached 11 g/dl, QALYs were incrementally reduced at treatment targets ≥11.5 g/dl.

**Table 4:** One-way sensitivity analyses of cost-effectiveness for different anemia treatment targets.

	ICER ($/QALY)						
Target Hb (g/dl)	10.0	10.5	11.0	11.5	12.0	12.5	13.0
Sensitivity Test							
Base Case	32,111	32,475	-	Dominated	Dominated	Dominated	Dominated
Stroke HR for ESA -50%	32,324	32,730	1,116,442	Dominated	Dominated	Dominated	Dominated
Stroke HR for ESA -100%	32,252	32,500	473,415	Dominated	Dominated	Dominated	Dominated
Stroke HR for Hb -50%	32,486	33,166	Dominated	Dominated	Dominated	Dominated	Dominated
Stroke HR for Hb -100%	32,531	33,405	Dominated	Dominated	Dominated	Dominated	Dominated
Stroke HR for Hb and ESA -50%	32,453	33,004	1,249,594	Dominated	Dominated	Dominated	Dominated
Stroke HR for Hb and ESA -100%	32,391	32,923	604,508	Dominated	Dominated	Dominated	Dominated
Non-CVD Mortality HRs for ESA -50%	32,936	34,350	176,275	1,071,379	Dominated	Dominated	Dominated
Non-CVD Mortality HRs for ESA -100%	33,607	35,806	106,395	169,753	270,993	600,458	Dominated
Non-CVD Mortality HRs for Hb -50%	27,747	31,871	Dominated	Dominated	Dominated	Dominated	Dominated
Non-CVD Mortality HRs for Hb -100%	Dominated	Dominated	Dominated	Dominated	Dominated	Dominated	Dominated
Non-CVD Mortality HRs for Hb and ESA -50%	29,298	34,699	Dominated	Dominated	Dominated	Dominated	Dominated
Non-CVD Mortality HRs for Hb and ESA -100%	Dominated	31,040	1,197,371	Dominated	Dominated	Dominated	Dominated
Hypertension HR for ESA -50%	32,366	32,839	Dominated	Dominated	Dominated	Dominated	Dominated
Hypertension HR for ESA -100%	32,368	32,803	27,896,637	Dominated	Dominated	Dominated	Dominated
QALY decrement for Hb -100%	38,591	43,388	Dominated	Dominated	Dominated	Dominated	Dominated
Cost of ESA +50%	34,086	39,460	Dominated	Dominated	Dominated	Dominated	Dominated
Cost of ESA -50%	30,647	26,254	Dominated	Dominated	Dominated	Dominated	Dominated
Start Treatment at Hb = 9	43,160	Dominated	Dominated	Dominated	Dominated	Dominated	Dominated
Start Treatment at Hb = 11	32,366	32,857	78,919	4,628,446	Dominated	Dominated	Dominated

CVD, cardiovascular disease; ESA, erythropoietin stimulating agents; Hb, hemoglobin; HR, hazard ratio; ICER, incremental cost-effectiveness ratio; QALY, quality adjusted life years.

**Fig 3:**
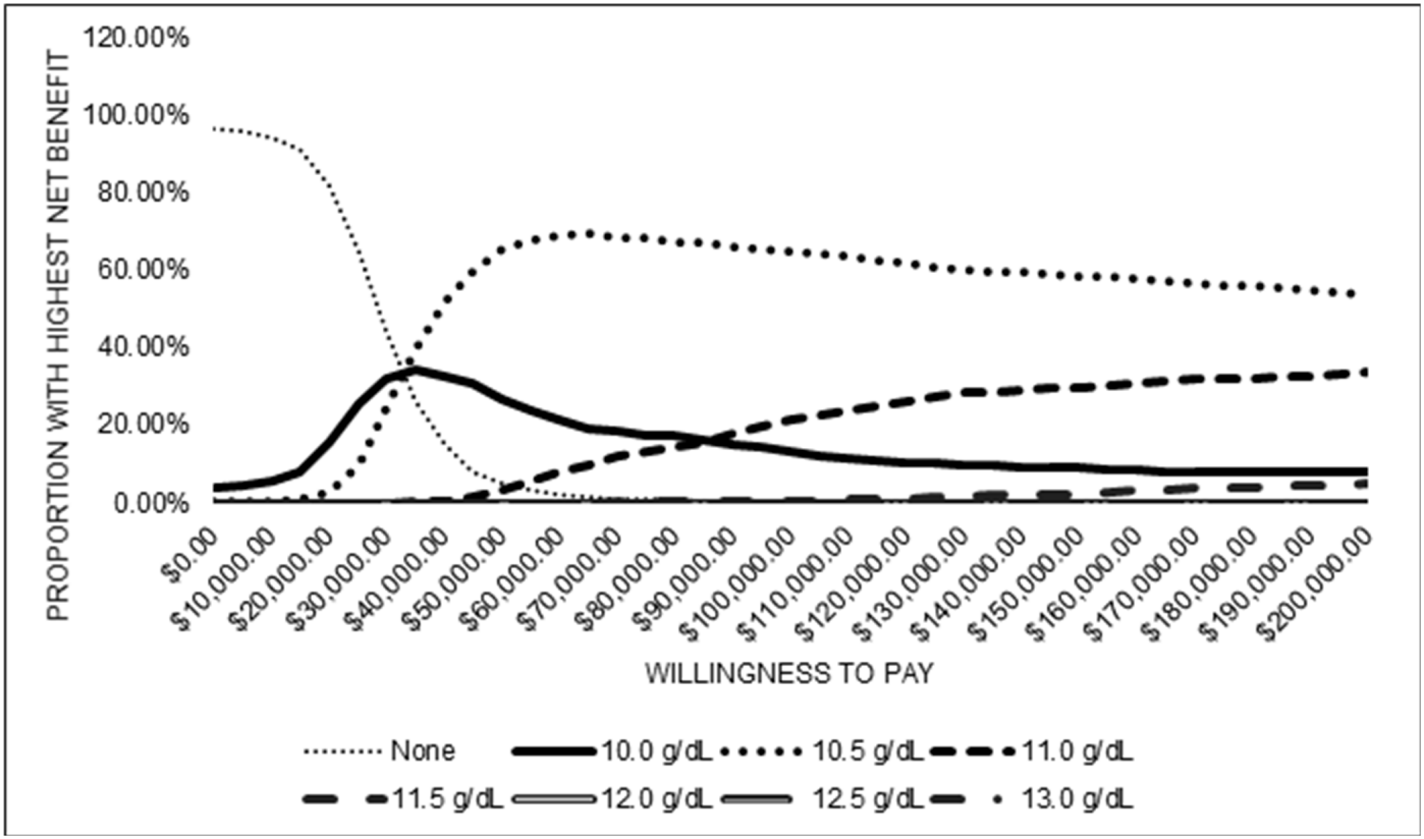
Cost-effectiveness acceptability curves from probabilistic sensitivity analysis of incremental cost-effectiveness for anemia treatment targets. Targets of 12.0 g/dl, 12.5 g/dl, and 13 g/dl never had the highest net benefit for any willingness to pay and therefore the corresponding curves equal the zero line.

Probabilistic sensitivity analysis results indicate ranges in costs, dosage, life years, stroke, and QALYs (Table 5). Median ICERs for Hb targets of 10 g/dl and 10.5 g/dl were positive, median ICER for a target of 11 g/dl was undefined, and median ICERs for Hb targets higher than 11 g/dl were dominated because those targets led to incrementally lower QALYs and higher costs. Cost-effectiveness acceptability curves show the distribution of the fraction of draws where the net benefits of each Hb target dominated (Fig 3). For low levels of willingness to pay, the no treatment strategy had the greatest proportion of draws with the highest net benefit. This proportion declined as willingness to pay increased and at a willingness to pay of approximately $30,000, a target of 10.5 g/dl became the strategy with the greatest proportion of draws with highest net benefit. A treatment target of 10.5 g/dl had the greatest proportion of draws with the highest net benefit for all higher levels of willingness to pay. The S1 Fig shows scatter plots of the incremental changes in costs and QALYs from the probabilistic sensitivity analysis for each Hb target.

**Table 5:** Median (95% CI) lifetime cost, ESA dosage, life years, QALYs, and ICERs from probabilistic sensitivity analysis of incremental cost-effectiveness for different anemia treatment targets.

Target Hb (g/dl)	Lifetime Costs ($)	ESA Dosage (units/week)	Life Years	QALYs	ICER ($/QALY)
No treatment	92,219 (88,264–97,651)	NA	6.46 (6.20–6.72)	4.47 (4.31–4.64)	NA
10.0	98,616 (94,121–103,424)	447 (421–472)	6.75 (6.50–7.02)	4.64 (4.48–4.81)	30,480 (-54,890–106,346)
10.5	102,750 (98,028–107,875)	1,232 (1,201–1,265)	6.92 (6.63–7.20)	4.76 (4.58–4.94)	30,059 (119,348–221,861)
11.0	108,540 (103,633–15,459)	3,873 (3,785–3,965)	6.88 (6.61–7.14)	4.76 (4.60–4.94)	-
11.5	115,255 (110,213–20,290)	7,476 (7,324–7,642)	6.75 (6.49–7.01)	4.71 (4.53–4.87)	Dominated
12.0	123,360 (117,728–28,864)	11,907 (11,683–12,134)	6.57 (6.29–6.83)	4.61 (4.43–4.77)	Dominated
12.5	132,372 (126,408–38,023)	17,221 (16,940–17,513)	6.33 (6.09–6.58)	4.46 (4.30–4.63)	Dominated
13.0	141,300 (135,035–47,232)	23,492 (23,163–23,839)	6.01 (5.77–6.24)	4.24 (4.08–4.40)	Dominated

CI, confidence interval; ESA, erythropoietin stimulating agents; Hb, hemoglobin; ICER, incremental cost-effectiveness ratio; QALY, quality adjusted years of life; NA, not applicable.

## Discussion

The present simulation modeling, employed to examine the cost-effectiveness of recommended anemia treatment targets, indicates that among persons with CKD stage 3–4 both Hb targets of 10 g/dl and 10.5 g/dl are cost effective, and the latter may be the most cost-effective. Targeting a Hb level of 11 g/dl increases costs without changes in QALYs when compared with a Hb level of 10.5 g/dl. Targeting Hb levels of 11 g/dl, 11.5 g/dl, or 12 g/dl increased QALYs compared with the base scenario of no treatment, but reduced QALYs when compared to next lower Hb treatment targets. These observations emphasize the importance of examining the incremental change in costs and QALYs in addition to the clinical diagnosis and management of persons with CKD when clinical practice guidelines are being developed. Past clinical guidelines have not considered incremental cost-effectiveness when recommending Hb treatment targets [3–5].

The present results have similarities and differences with past studies examining the cost-effectiveness of anemia treatment for persons with CKD. Clement et al. used a simulation model to examine the cost-effectiveness of four alternative Hb treatment targets (no ESA treatment, 9–10.9 g/dl, 11–12 g/dl, and > 12 g/dl) in a cohort of Canadian persons with CKD stage 4 or higher including those on dialysis [32]. This study reported similar results to the present analysis: a treatment target between 9 g/dl and 10.9 g/dl increased QALYs and costs, but Hb targets between 11 g/dl and 12 g/dl and between 12 g/dl and 13 g/dl reduced QALYs and increased costs. However, direct comparison between the Clement et al. study and the current study is not fully possible because Clement et al. did not examine specific Hb levels within each range. Quon et al. used a simulation model to examine the cost-effectiveness of two alternative Hb treatment target ranges (10–11 g/dl and 9–10 g/dl) in the US hemodialysis population [33]. The study found that a treatment target of 10–11 g/dl increased QALYs and reduced costs compared with a target between 9 g/dl and 10 g/dl. However, Hb targets above 11 g/dl and specific Hb levels within each range, were not examined, so it is not clear how the results from Quon et al. fit into the broader range of Hb treatment target possibilities. Tonelli et al. used a simulation model to examine the cost-effectiveness of four treatment targets (9.5–10.5 g/dl, 11–12 g/dl, 12–12.5 g/dl, and 14 g/dl) in the US dialysis population [34]. The study found that QALYs increased with increasing Hb levels up to 14 g/dl and that a target range of 11–12 g/dl was cost effective while the two higher targets (12–12.5 g/dl and 14 g/dl) were not. This study by Tonelli et al. was published in 2003 and was based on earlier assumptions about the benefits of increasing Hb, which did not incorporate complications from higher ESA dose that are included in our study.

Differences with past studies may be driven by different study populations and different Hb treatment targets examined. Past studies have all included dialysis populations, which may have led to different results. Past studies have also grouped together relatively large Hb ranges when examining alternative treatment targets. This can impact results because ICERs are sensitive to the comparison Hb target. For example, in the present study, treatment targets that are dominated when compared to the next lower treatment target would be considered cost-effective if compared to the no ESA treatment scenario.

One of the innovations of our model is the integration of emerging research indicating a risk-benefit tradeoff associated with increasing ESA dosage. We chose this approach because recent published research has found that while higher Hb levels are associated with reduced complications, the higher ESA dose needed to achieve those Hb levels is associated with increased drug-related complications, including stroke and non-CVD mortality [14, 27–29]. This approach allows for the replication of the relationship between target Hb level and outcome observed when comparing recent large clinical trials such as CHOIR [24], CREATE [25], and TREAT [26]. Comparing these large trials produces an inverted-u shaped relationship between target Hb level and QALYs. Fig 2 above, demonstrates this same relationship in the model. This ability to replicate the relationship seen in large clinical trials gives confidence in model results. Past cost-effectiveness studies have not reproduced this relationship well.

Our study is subject to four limitations. First, the model relies on available published research, so although model parameters were based on the current state of the literature, they may be imperfect or may omit additional unknown factors such as factors related to other potential anemia complications such as heart failure. For example, the quality of life parameters for Hb level came from a cross-sectional study [16] and had to be mapped to QALYs using a mapping function [17], both of which pose limitations. This makes it essential to test the sensitivity of results to parameter choices. Second, the model is based on the non-dialysis population over age 30 with CKD stages 3 or 4 and anemia at baseline, so results may not be generalizable to other populations such as those younger than age 30 and those on dialysis. Third, the model does not include acute kidney failure. Finally, it is important to emphasize that this is a simulation modeling study and optimally, the results would be validated in a controlled trial.

The present analysis sheds light on the cost-effectiveness of alternative Hb treatment targets and indicates that achieving a target Hb of 10.5 g/dl is most cost-effective in a cohort of people with CKD stages 3–4. This is an important step in informing and framing future guidelines around the treatment of anemia and in improving outcomes in persons with moderate to advanced CKD. It also serves as a model for testing the cost-effectiveness of other treatment interventions in people with CKD.

## Supporting Information

S1 Appendix Estimation of Select Anemia Module Parameters. (DOCX)

S1 Fig Distribution of incremental costs and QALYs in probabilistic sensitivity analysis by treatment target. (A) Hb target = 10 g/dl; (B) Hb target = 10.5 g/dl; (C) Hb target = 11 g/dl; (D) Hb target = 11.5 g/dl; (E) Hb target = 12 g/dl; (F) Hb target = 12.5 g/dl; (G) Hb target = 13 g/dl.
(TIF)
