# Supplementary material for: The Cost-Effectiveness of Anemia Treatment for Persons with Chronic Kidney Disease
Source: PLoS One. 2016 Jul 12;11(7):e0157323. doi: 10.1371/journal.pone.0157323 (PMC4942058; doi:10.1371/journal.pone.0157323)
Supplement: S1 Appendix — (DOCX) [file pone.0157323.s001.docx]

**S1 Appendix: Estimation of Select Anemia Module Parameters**

**Modeled Anemia Status and Hemoglobin Level**

The model assigns a level of hemoglobin annually based on the hemoglobin distribution in the National Health and Nutrition Examination Survey (NHANES) and the agent’s risk factors (age, race, diabetes, cardiovascular disease [CVD], chronic kidney diseases [CKD] stage, and estimated glomerular filtration rate [eGFR]). We begin with the standardized (i.e., mean 0 and standard deviation 1) hemoglobin distribution, by risk factor from NHANES. Each individual was assigned a standardized hemoglobin value that represents their position in the distribution. We then used NHANES data to regress anemia status on risk factors, used the coefficients (Table S1-1) to predict the probability of anemia, and generated a distribution for the predicted probability of anemia by all risk factor combinations. Using the same data, we regressed hemoglobin level on risk factors and anemia status. We used the coefficients (Table S1-2) to predict hemoglobin level and generated the distribution of predicted hemoglobin level for all risk factor combinations.

We generated hemoglobin levels for each agent using the standardized hemoglobin distribution and these predictions. We did this differently by CKD stage:

- eGFR > 60 (i.e. normal or CKD stage 1 or 2): Agents are assumed not to have anemia. hemoglobin = agent’s standardized hemoglobin value * standard deviation of hemoglobin prediction + mean of hemoglobin prediction for risk factor group without anemia.
- CKD Stage 3: We assign an agent to anemia or no anemia randomly based on their predicted probability of anemia. In an agent’s first simulated year in stage 3, their hemoglobin value is generated from their standardized hemoglobin value and the predicted hemoglobin value that is based on risk factors and assigned anemia status. After the initial year in stage 3, changes in hemoglobin level are based on changes in risk factors and regression coefficients.
- CKD Stage 4: If the agent enters stage 4 without anemia, we assign them to anemia or no anemia randomly based on their predicted probability of anemia. If an agent enters stage 4 directly at model start, then initial hemoglobin is generated as in stage 3. If an agent already has anemia when entering stage 4, their hemoglobin level carries over. Changes in hemoglobin level are based on changes in risk factors and regression coefficients.
- CKD Stage 5: If the agent enters stage 5 without anemia, we assign them to anemia or no anemia randomly based on their predicted probability of anemia. If an agent enters stage 5 directly at model start, then initial hemoglobin is generated as in stage 3. If an agent already has anemia when entering stage 5, their hemoglobin level carries over. Changes in hemoglobin level are based on changes in risk factors and regression coefficients.

**Table S1-1. Anemia Status Logit Regression Coefficients**

| Variable | Males | Females |
| --- | --- | --- |
|  | Coefficient | Coefficient |
| Black (reference group = white) | 1.23 | 1.46 |
| Ages 50–64 (reference group = age 30-49) | 1.97 | −0.95 |
| Ages 65+ (reference group = age 30-49) | 2.48 | −1.10 |
| Diabetes (reference group = no diabetes) | −0.18 | 0.61 |
| CVD (reference group = no CVD) | −0.27 | 0.12 |
| CKD Stage 3 (reference group = CKD less than stage 3 or no CKD) | 1.61 | 0.87 |
| CKD Stage 4 (reference group = CKD less than stage 3 or no CKD) | 3.47 | 2.80 |
| Constant | −7.63 | −3.67 |

**Table S1-2. Hemoglobin OLS Regression Coefficients**

| Variable | Males | Females |
| --- | --- | --- |
|  | Coefficient | Coefficient |
| Anemia (reference group = no anemia) | −4.29 | −3.37 |
| Black (reference group = white) | −0.78 | −0.73 |
| Ages 50–64 (reference group = age 30-49) | −0.15 | 0.27 |
| Ages 65+ (reference group = age 30-49) | −0.59 | 0.17 |
| Diabetes (reference group = no diabetes) | −0.28 | −0.14 |
| CVD (reference group = no CVD) | −0.17 | −0.04 |
| CKD Stage 3 (reference group = CKD less than stage 3 or no CKD) | −0.39 | −0.20 |
| CKD Stage 4 (reference group = CKD less than stage 3 or no CKD) | −1.04 | −0.96 |
| Constant | 15.57 | 13.69 |

**Imputation of Non-CVD Hazard Ratios**

We generated hazard ratios that modify the risk of non-CVD mortality. This hazard ratio is a function of hemoglobin level and dose of ESA. We did not have a direct source for non-CVD mortality associated with these two factors, so we imputed the hazard ratios using hazard ratios for CVD mortality and all-cause mortality taken from Koulouridis et al. (2013). We approximate non-CVD mortality by solving the following equation:

HRanycause = θ HRCVD + (1-θ) HRnonCVD

where θ, the share of CVD deaths, is 1/3.

**ESA Dose**

Anemia in chronic kidney disease (CKD) is treated primarily with erythropoietin stimulating agent (ESA) drugs. In our model, we focus on one of these drugs, epoetin alfa. The alternative drug, darbepoetin alfa, is a comparable substitute. To determine the epoetin alfa dose necessary to achieve a given change in hemoglobin, we followed the approach of past simulation studies (Quon et al., 2012; Tonelli et al., 2003) and fit a regression equation to data on epoetin alfa dose and change in hemoglobin from clinical trials (taken from Koulouridis et al. 2013). We stratified this analysis by baseline hemoglobin to allow for different dosage requirements for different baseline levels of hemoglobin. Because the model logically must have a zero weekly dose for a target change in hemoglobin of zero, we did not include a constant term or a level effect term for baseline Hb. Specifically, we estimated the following regression equation:

$$Weekly Dose= \beta_{1}\Delta Hb+\beta_{2}{\Delta Hb}^{2}{+\beta}_{4}\Delta Hb*BaselineHb+\beta_{5}{\Delta Hb}^{2}*BaselineHb$$

Agents are assigned a dose based on their target change in hemoglobin, baseline hemoglobin, and the coefficients from this regression (Table S1-3).

Table S1-3. Coefficients for Weekly Epoetin Alfa Dose

| Variable | Coefficient |
| --- | --- |
| ΔHb | -1,204 |
| ΔHb^2^ | -1,365 |
| ΔHb*Baseline Hb | -41.76 |
| ΔHb^2^*Baseline Hb | 436 |
