# Supplementary figures and images for: The Cost-Effectiveness of Anemia Treatment for Persons with Chronic Kidney Disease
Source: PLoS One. 2016 Jul 12;11(7):e0157323. doi: 10.1371/journal.pone.0157323 (PMC4942058; doi:10.1371/journal.pone.0157323)

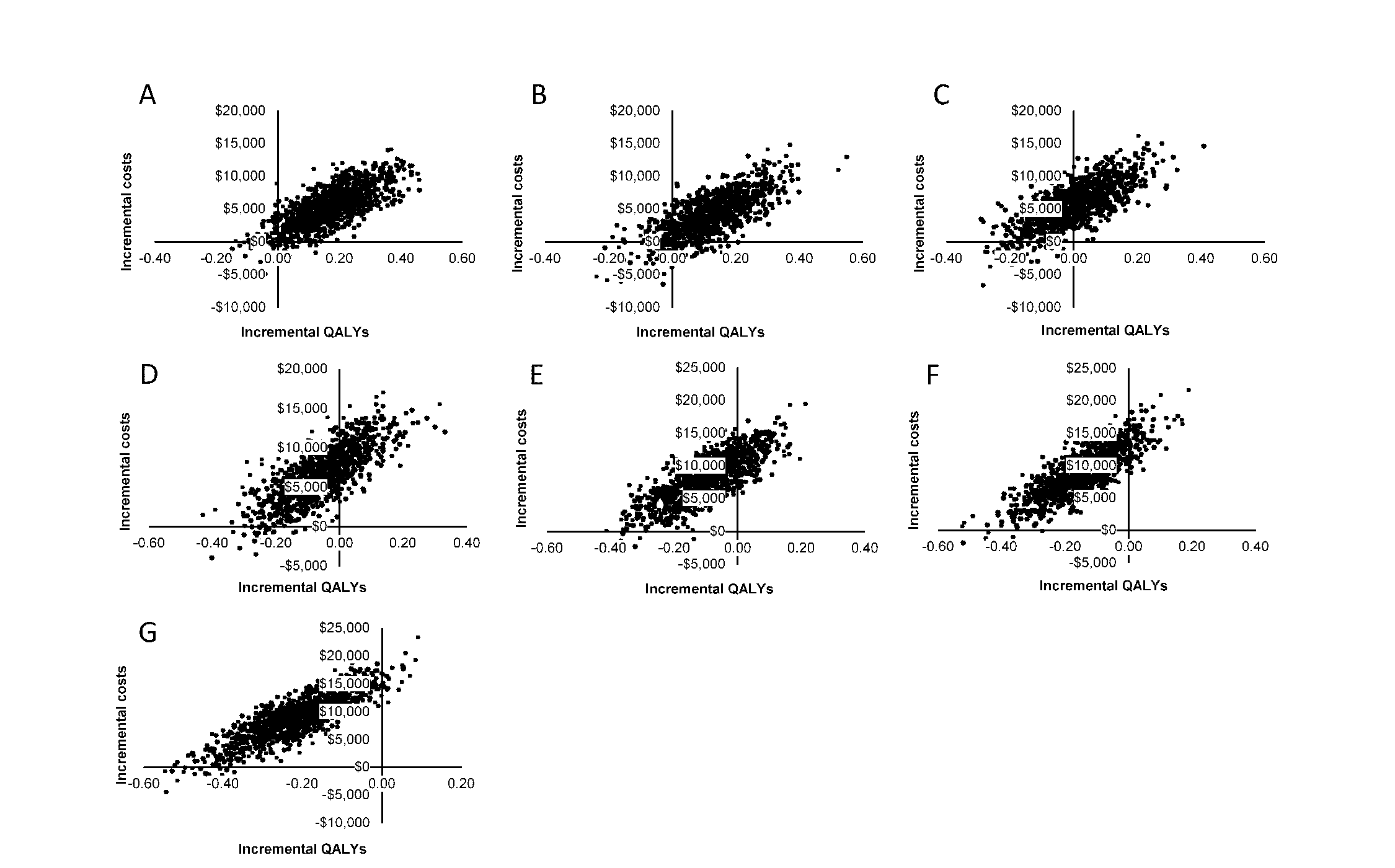

Supplement: S1 Fig — (A) Hb target = 10 g/dl; (B) Hb target = 10.5 g/dl; (C) Hb target = 11 g/dl; (D) Hb target = 11.5 g/dl; (E) Hb target = 12 g/dl; (F) Hb target = 12.5 g/dl; (G) Hb target = 13 g/dl. (TIF) [file pone.0157323.s002.tif]
